# Viscoelastic Oxidized Alginates with Reversible Imine Type Crosslinks: Self-Healing, Injectable, and Bioprintable Hydrogels

**DOI:** 10.3390/gels4040085

**Published:** 2018-11-21

**Authors:** Shahzad Hafeez, Huey Wen Ooi, Francis L. C. Morgan, Carlos Mota, Monica Dettin, Clemens van Blitterswijk, Lorenzo Moroni, Matthew B. Baker

**Affiliations:** 1 Department of Complex Tissue Regeneration, MERLN Institute for Technology Inspired Regenerative Medicine, Maastricht University, P.O. Box 616, 6200 MD Maastricht, The Netherlands; s.hafeez@maastrichtuniversity.nl (S.H.); h.ooi@maastrichtuniversity.nl (H.W.O.); f.morgan@maastrichtuniversity.nl (F.L.C.M.); c.mota@maastrichtuniversity.nl (C.M.); c.vanblitterswijk@maastrichtuniversity.nl (C.v.B.); 2 Department of Industrial Engineering, University of Padua, 35131 Padua, Italy; monica.dettin@unipd.it

**Keywords:** dynamic hydrogel, dynamic covalent chemistry (DCvC), reversible bonds, bioprinting (BP), oxidized alginate (ox-alg), tissue engineering, viscoelastic, hydrazone, semicarbazone, oxime

## Abstract

Bioprinting techniques allow for the recreation of 3D tissue-like structures. By deposition of hydrogels combined with cells (bioinks) in a spatially controlled way, one can create complex and multiscale structures. Despite this promise, the ability to deposit customizable cell-laden structures for soft tissues is still limited. Traditionally, bioprinting relies on hydrogels comprised of covalent or mostly static crosslinks. Yet, soft tissues and the extracellular matrix (ECM) possess viscoelastic properties, which can be more appropriately mimicked with hydrogels containing reversible crosslinks. In this study, we have investigated aldehyde containing oxidized alginate (ox-alg), combined with different cross-linkers, to develop a small library of viscoelastic, self-healing, and bioprintable hydrogels. By using distinctly different imine-type dynamic covalent chemistries (DCvC), (oxime, semicarbazone, and hydrazone), rational tuning of rheological and mechanical properties was possible. While all materials showed biocompatibility, we observed that the nature of imine type crosslink had a marked influence on hydrogel stiffness, viscoelasticity, self-healing, cell morphology, and printability. The semicarbazone and hydrazone crosslinks were found to be viscoelastic, self-healing, and printable—without the need for additional Ca^2+^ crosslinking—while also promoting the adhesion and spreading of fibroblasts. In contrast, the oxime cross-linked gels were found to be mostly elastic and showed neither self-healing, suitable printability, nor fibroblast spreading. The semicarbazone and hydrazone gels hold great potential as dynamic 3D cell culture systems, for therapeutics and cell delivery, and a newer generation of smart bioinks.

## 1. Introduction

As we strive to recapitulate the complexity of native tissues and organs, bioprinting (BP) has emerged as a promising group of technologies for fields ranging from fundamental cell biology to clinically relevant tissue engineering [1]. BP allows the printing of various materials, cells, and biological molecules in defined space. These technologies have allowed progress toward the custom 3D construction of both soft and hard tissues including skin, heart, kidney, and bone [1,2]. Bioprinting of high modulus materials (> 1 MPa range) for hard tissues, such as bone [3], has already been successfully demonstrated on a clinical scale for a wide range of polymeric materials (e.g., polyurethane, polycaprolactone, and block copolymers such as PEOT/PBT [4,5]) and printing techniques (e.g., 3D plotting [6] and fused deposition modelling [7]). However, the bioprinting of biological constructs for softer tissues with complex architecture remains limited. Though several key studies have recently shown strategies to design hydrogels with enhanced capacity to self-support bioprinted structures for soft tissues [8,9,10], the demand for customizable bioinks with tissue relevant viscoelasticity and shear-thinning properties required for bioprinting remains unfulfilled.

Bioprinting of cell-laden hydrogels (bioinks) is attractive for the construction of complex soft tissue architectures, allowing for the creation of constructs with highly defined placement of both material composition and cells [5,11]. However, printing with live cells comes with a unique set of challenges for the hydrogel system used. An ideal bioink is printable, tailorable mechanically and chemically, and maintains structural integrity—all while maintaining high cell viability and mimicking the complex mechano-chemical signals of the native extracellular matrix (ECM) [12]. Several successful material formulations have been developed with unique combinations of polymers (e.g., alginate [13,14], gelatin [15], gelatin methacrylate [16], hyaluronic acid [17], and polyglicidol [18,19]) and crosslinking strategies (ionic [13], Schiff base [15], free-radical [16,20], thiol-ene [19,21,22], and supramolecular [9]). While significant progress has been made in bioink development, only a few of these materials show viscoelasticity comparable to soft tissue [23,24,25].

Recent studies have shown the important effects a viscoelastic material can have on cell behavior and tissue formation. For example, a material’s viscoelastic timescale can influence spreading and proliferation of adherent cells in hydrogels [26,27,28] and enhance the differentiation of mesenchymal stem cells (MSCs), leading to more advanced bone tissue formation [29]. Chondrocytes in viscoelastic hydrogels have also been shown to effectively deposit the cartilage matrix [30]. A common approach to facilitate the design of viscoelastic hydrogels are networks consisting of reversible bonds. These reversible bond systems have also been shown to facilitate adherent cell spreading [27,31], myoblasts fusion [24], and MSC differentiation into multiple lineages [32]. Owing to their reversible dynamic bonds, such materials can respond to cell forces via network rearrangement, breaking and reforming the bonds, while keeping bulk biophysical properties constant [9,25,33].

In an effort to move towards smarter materials for BP, a conceptually appealing approach is to utilize gels with reversible bonds and dynamic interactions. Such systems not only allow for tunable viscoelastic properties of cell-laden constructs, but also for the incorporation of shear-thinning behavior, reducing viscosity and increasing printability when a shear stress is applied. Consequently, efforts have already been made to develop viscoelastic materials with shear-thinning and self-healing properties for BP. A prominent example is the hyaluronic acid hydrogels based on supramolecular host-guest complexation (adamantane and cyclodextrin). The shear-thinning and self-healing nature of this hydrogel allow for gel in gel bioprinting, yet generally requires a secondary crosslinking step to allow free standing structures is limited in its ability to support cell adhesion [17,34]. 

Dynamic covalent chemistry (DCvC) provides a large tool-box for the construction of dynamic soft materials [23,33]. Imine type DCvCs are particularly attractive as they possess a range of equilibrium contents (K_eq_), and tunable hydrolysis rates at physiological pH (*k*_−1_) have been widely explored for bio-conjugation strategies, and are generally chemically specific and orthogonal. With the knowledge of the K_eq_ and *k*_−1_ of different imine linkages, one can tune the physical properties of a gel. In general, K_eq_ affects the stiffness of the gel, while *k*_−1_ can be used to tune the rate of crosslink rearrangement and the viscoelastic response. The rate of hydrolysis (*k*_−1_) for different imine type crosslinks have previously been shown to decrease with increasing electronegativity of the group alpha to the primary amine: Setting oxime hydrolysis at one, semicarbazone hydrolyzes 160 times faster, and hydrazone 300 times faster under identical conditions [35]. Conveniently, imine formation and its hydrolysis is readily tunable based on the type of imine bond formed [36], and can be highly sensitive to pH [35,37,38]. Imine type reversible covalent viscoelastic hydrogels as 3D cell culture platforms that are self-healing, shear-thinning, and injectable have also been developed [24,25,32]. However, there have been only a few studies exploring DCvC for BP [8,10].

**Figure 1:**
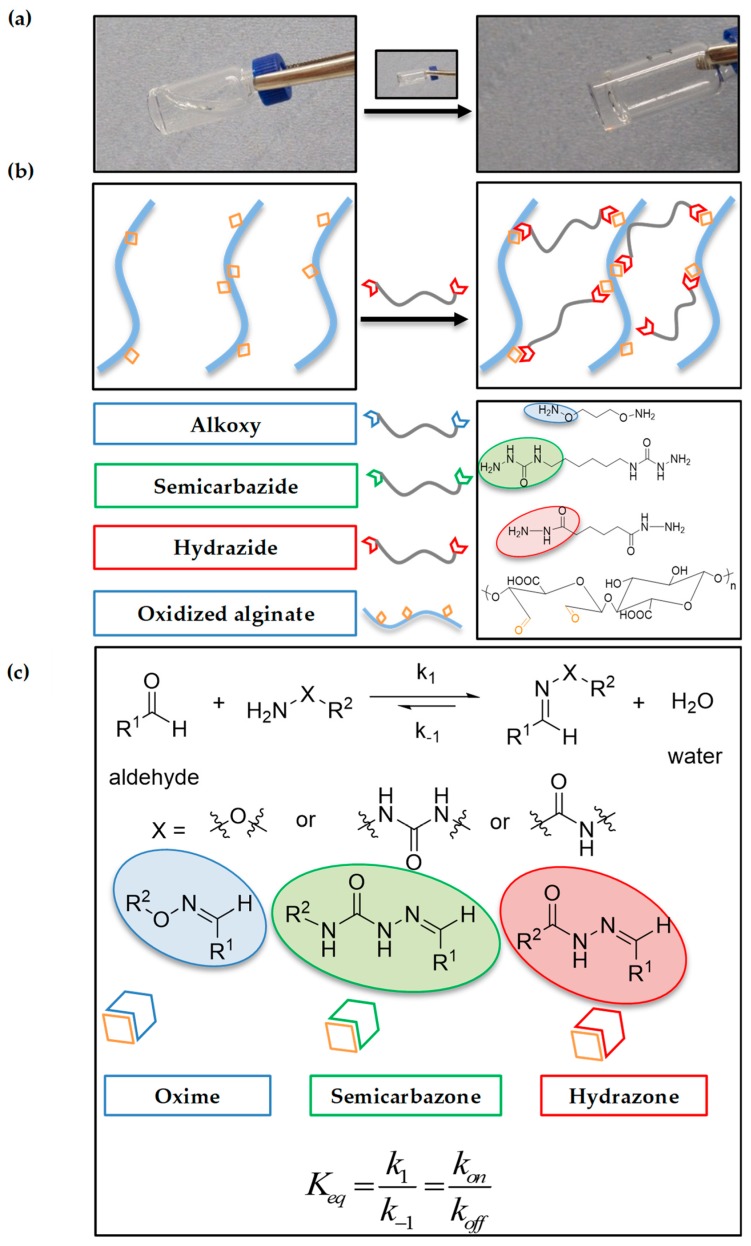
Scheme of hydrogel formation. (**a**) Representative gel formation for a hydrazone gel. (**b**) Schematic illustrating the crosslinking of alginate chains using dihydrazide cross-linker, the structure of oxidized alginate and the structures of cross-linkers utilized in this study. (**c**) A general reaction scheme for reversible imine type bonds formation and chemical structures of oxime, semicarbazone and hydrazones.

In light of the recent importance of reversible hydrogel networks (e.g., viscoelasticity and stress relaxation) within cell culture and tissue formation [24,29,31], we aimed to create a 3D printable bioink that better recapitulates these ECM inherent properties. We hypothesized that alginate hydrogels with different imine type crosslinks (oxime, semicarbazone, and hydrazone shown in Figure 1) would give rise to gels with different viscoelasticity, self-healing and shear-thinning properties, resulting in different printability. Alginate, a naturally derived polysaccharide composed of β-d-mannuronic acid (M units) and α-l-guluronic acid (G units), is economically obtainable and has a long history in tissue engineering and bioprinting [39,40]. Alginate is traditionally rapidly cross-linked via divalent ions (e.g., Ca^2+^) and has high biocompatibility, high viscosity, low gelation concentrations, and no specific interactions with cells [41,42], yet the bioprintablity of unmodified alginate provides relatively poor performance due to the stiffness and viscosity tradeoff between cell viability and printability [12]. The oxidation of alginate, via the introduction of aldehyde groups along the backbone [43], can increase its biodegradability and has been previously leveraged for tailorable hydrogel formation via imine type crosslinking in drug delivery [44,45]. Despite the power of this system, oxidized alginate cross-linked by imine bonds has only been partially explored, and to the best of our knowledge, has never been reported in the context of bioprinting.

In this study, we found that oxidized alginates cross-linked with different imine-type bonds allow an easily obtainable and modifiable bioink platform for 3D printing. The gels created are mechanically tunable, biocompatible, shear-thinning, self-healing, and preserve cell viability post-extrusion. Furthermore, these gels are biofunctionalizable via traditional oxime ligation strategies, and show interesting changes in cell adhesion as a function of the dynamic bond used. Integrating tunable viscoelasticity into a bioink formulation brings us one step closer to the creation of synthetically modifiable ECM-mimicking hydrogels for bioprinting. Their unique materials properties, along with the ability to tune the mechanochemical signals, will allow for the modular construction of more complex soft tissue mimics in the future.

## 2. Results and Discussion

### 2.1. Alginate Oxidation and Model Reactions

Oxidation of alginate was carried out using sodium periodate (NaIO_4_) as an oxidizing agent, and according to literature procedures [40]. The hydroxyl groups at C-2 and C-3 of the repeating uronic acid units were oxidized resulting in the breakage of the C–C bonds to form two aldehyde groups, rapidly forming hemicacetals with neighboring alcohol groups [46]. Controlling the stoichiometric addition of NaIO_4_, sugar monomers in alginate chains were oxidized to 5% (5% ox-alg), 10% (10% ox-alg), and 15% (15% ox-alg) theoretical degrees of oxidation (DOX) and confirmed qualitatively with the appearance of increasing hemiacetal peaks in NMR spectra (Figure S3 in Supplementary Materials). Subsequently, model reactions with small molecules were performed to investigate the possibility of forming hydrazone, semicarbazone, and oxime linkages within a reasonable time scale. At pH 7.4 and room temperature (RT), bond formation after 30 min—relevant conditions for formation of cell-encapsulated hydrogels—was monitored via NMR. The appearance of imine type bonds (oxime, semicarbazone, and hydrazone) were confirmed with ^1^H NMR spectroscopy (Figure S4 in Supplementary Materials). The concurrent appearance of imine type hydrogens (6.5–8.5 ppm) and reduction in the hemiacetal hydrogens (5.15–5.75 ppm) provide evidence that the aldehydes along the oxidized alginate backbone could undergo the imine type bond formation relatively quickly under suitable conditions.

### 2.2. Hydrogel Formation

Next, we evaluated the ability of three different cross-linkers designed to form imine type crosslinks (adipic acid dihydrazide, hexamethylene disemicarbazide, and aminooxy propyl hydroxyl amine dihydrochloride) to form a gel at RT in phosphate buffered saline (PBS) at pH 7.4 using 15% ox-alg, 2% (*w*/*v*) and an equimolar concentration of cross-linker (to the theoretical oxidation degree). All cross-linkers successfully made self-standing hydrogels (hydrazone gel is shown in Figure S8 in Supplementary Materials). However, gel formation kinetics for each linker were slightly different. Monitored visually via vial inversion, the oxime crosslinks were found to have the fastest kinetics (≈10 min stable gel formation time) followed by semicarbazone (≈15 min stable gel formation time) and then hydrazone (≈45 min stable gel formation time). During these experiments, we also observed that oxime crosslinks were non-reversible (on the time scale of our experiment), while semicarbazone and hydrazone crosslinks were reversible (vide infra). 

**Figure 2:**
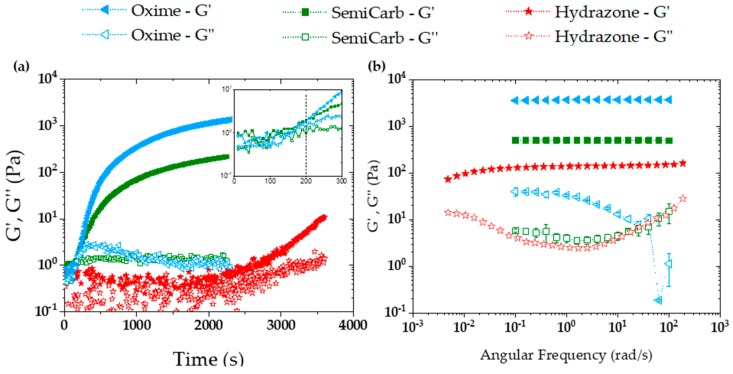
Hydrogel formation kinetics and viscoelasticity exhibited by a series of 2% (*w*/*v*) 10% ox-alg samples prepared with different cross-linkers (equimolar). (**a**) Time sweep using the three different cross-linkers; aminooxy, semicarbazide, and hydrazide for evaluating hydrogels formation kinetics measured at 1% strain and 10 rad/s. (**b**) Frequency sweep for the same three hydrogels measured at 1% strain.

To obtain more insight into hydrogel formation kinetics, the gelation time was observed via plate-plate rheology. A time sweep was recorded to determine the gelation point, defined here as the crossover point between G′ and G″, after which there was a sudden increase in G′. For example, for 10% ox-alg, a 2% *(w*/*v)* solution was briefly mixed with equimolar concentration of cross-linkers rheological measurements were started within four minutes. The oxime and semicarbazone hydrogels showed a G′ and G″ crossover point (gel point) around eight minutes, while the gel point was delayed until roughly 45 min for hydrazone. G′ continued to increase towards a plateau after approximately 30 min for oxime and semicarbazone; however, hydrazone showed a continuous increase in G′ even after 60 min (shown in Figure 2a). Rheological and vial inversion experiments clearly showed that imine type chemistry has an influence on gelation kinetics, with oxime and semicarbazone gelation significantly faster than hydrazone. 

Unless otherwise stated, in the remainder of this study, 10% ox-alg at 2% (*w*/*v*), and an equimolar amount of cross-linker (to the theoretical oxidation degree) were used to make gels.

### 2.3. Rheology

#### 2.3.1. Storage and Loss Moduli 

In order to determine the mechanical properties of the hydrogels, oscillatory shear rheology was performed. The mole equivalents of cross-linker, type of ox-alg, concentration % (*w*/*v*), and cross-linking time were kept constant to independently evaluate the effect of crosslinks on stiffness and viscoelasticity. The oxime, semicarbazone (denoted by SemiCarb in Figure 2), and hydrazone crosslinks form gels whose shear moduli decrease across the series. As seen in the frequency sweep plots (Figure 2b), the oxime gel was the stiffest (G′ ≈ 4000 Pa), followed by semicarbazone (G′ ≈ 500 Pa), and then hydrazone (G′ ≈ 200 Pa). The oxime gel was roughly eight and 20 times stiffer than semicarbazone and hydrazone, respectively, for an identical formulation.

For cross-linked polymer networks, shear modulus is directly proportional to the number of elastically active crosslinks [25] (Equation (1)),

(1) $$\;G^{\prime }\;=kT\frac{\nu_{e}}{\bar{V}}\;$$

where *k* is Boltzmann constant, *T* is the temperature, and *ν_e_* represents the number of elastically active crosslinks per unit volume ($\bar{V}$). In hydrogels with reversible crosslinks, the crosslinking density depends on the ratio of formed crosslinks to unbound crosslinks. Assuming similar topological architecture in the system, this difference in the amount of crosslinking can be correlated with the K_eq_ of the different systems; a system with a higher modulus should be a direct result of more crosslinks indicating a higher K_eq_ of the DCvC bond [24,33]. Our results would suggest approximately one order of magnitude more crosslinking going from the hydrazone to the semicarbazone, and from the semicarbazone to the oxime. This result is nicely aligned with the trend in equilibrium constants of model systems. Oximes generally have the highest K_eq_ (>10^8^ M^−1^) followed by semicarbazones and then hydrazones, which are in the range of 10^4^–10^6^ M^−1^ [35].

To investigate the effect of the degree of oxidation (DOX) on stiffness, 5% ox-alg, 10% ox-alg, and 15% ox-alg gels were made using the dihydrazide cross-linker. Generally, an increase in storage moduli was observed with increasing DOX. Storage moduli increased by a factor of four (from ~90 to ~350 Pa) as the DOX increased from 5% to 10%; however, no considerable difference was observed between 10 and 15% ox-alg (~400 Pa) (Figure S7 in Supplementary Materials). The observed increase in storage moduli has been correlated to the larger number of crosslinking sites available (aldehydes) with increasing DOX [43,46]. While we did not observe a significant difference between 10% and 15% ox-alg gels, the NMR of the base material (Figure S3) indicated the presence of more aldehydes for the 15% ox-alg.

#### 2.3.2. Viscoelasticity 

The frequency sweep (Figure 2b) for both the semicarbazone and the hydrazone gels showed characteristic viscoelastic behavior, with frequency dependent loss moduli over the range explored. While the oxime gel did show some frequency dependence, this appears to represent more elastic behavior. Viscoelastic materials can also show a crossover point (example in Figure S5) between storage (G′) and loss modulus (G″), while moving from rubbery plateau (higher frequency) to terminal region (low frequency). However, this crossover point was not observed even during a longer range scan of the hydrazone gel down to 0.005 rad/s.

The crossover point in these reversible bond systems is largely determined by the rate at which a bond breaks (*k*_−1_) which, for imine type bonds, is directly related to the reverse rate of hydrolysis (as the rate limiting step in the off reaction). From our observation of material behavior and moduli evolution, hydrazone should have the highest *k*_−1_, among all crosslinks, which is an indication that hydrazone gel will show a crossover point at a higher frequency (rad/s), as compared to semicarbazone and oxime. In Figure 2b, a decrease in the storage modulus (G′) and an increase in tanδ forecast a transition between rubbery and terminal regions and eventual convergence of G′ and G″. Typically, low frequencies are needed to observe a crossover point for hydrazone crosslinks; Anseth and co-workers have observed a crossover point around 0.03 rad/s for methyl hydrazone crosslinks on 4-arm PEG [24]. In this study, acyl hydrazone was utilized, which has half the hydrolysis rate (*k*_−1_) of methyl hydrazone. Therefore, convergence of G′ and G″ below 0.03 (rad/s) was expected and is not surprising for a linear alginate polymer. These results suggest that chain length, topology of the polymer (linear versus multi-arm), and functionalities for effective crosslinks are all critical factors to tune viscoelasticity. However, as such, a crossover point at such small frequencies suggests that the alginate imine cross-linked hydrogels are very slow stress relaxing networks.

#### 2.3.3. Self-Healing

Self-healing was initially visualized in the lab for the 15% ox-alg gel. In a glass vial, the gel network was broken using a spatula and self-healing was observed over time via the vial inversion method. The semicarbazone self-healed in ≈10 min, while hydrazone took ≈30 min to self-heal (Figure S8 in Supplementary Materials). The oxime gel did not self-heal. Oxime bonds are more stable than the semicarbazone and hydrazone bonds as the aminooxy is more electron deficient compared to the hydrazides. Presumably, the enhanced stability (kinetically and thermodynamically) at neutral pH makes the reversibility/exchange dynamics too slow for oxime, resulting in no self-healing at room temperature and neutral pH [35,47].

**Figure 3:**
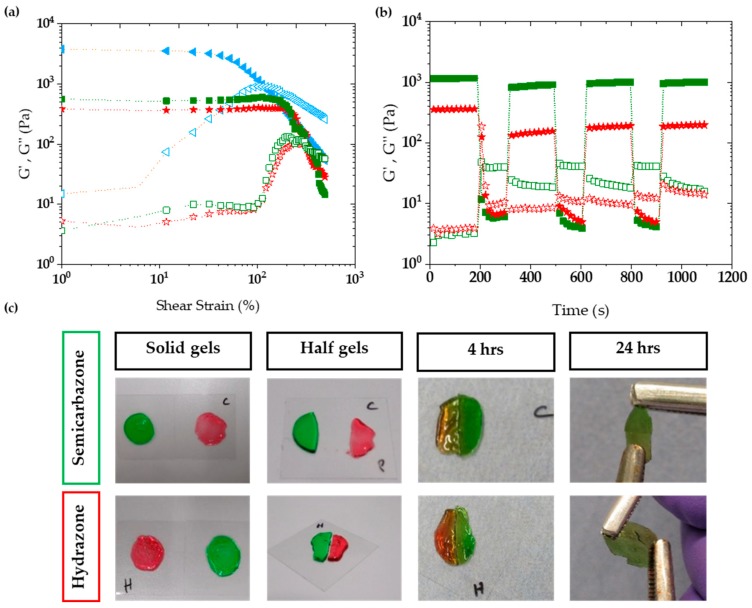
Demonstration of strain to rupture the network and the self-healing capacity of 10% ox-alg samples prepared with semicarbazide and hydrazide cross-linkers. (**a**) Strain sweeps from 0% to 800% strain using oxime, semicarbazone, hydrazone and hydrogels, measured at 10 rad/s. (**b**) self-healing capacity with semicarbazide and hydrazide cross-linkers. The hydrogels were submitted to three strain cycles. Initially a low strain (1%) was applied, followed by three cycles of high strain (600%), to rupture the network, and low strain (1%), to allow recovery. The semicarbazone and hydrazone gels rapidly self-heal. (**c**) Macroscopic self-healing of the hydrogels. From left to right, the colored gels were formed (12 mm diameter, 2 mm thickness), cut in half, left to self-heal for four hours, and then tested at 24 h.

In order to investigate the self-healing behavior more thoroughly, shear rupture-self-healing cycles were carried out on the rheometer. Initially, strain sweep experiments were carried out to determine the amount of strain needed for gel rupture. These gels proved to be surprisingly tough, with the crossover point for all gels being between 100% and 400% strain (shown in Figure 3a and Figure S6). Thus, 600% strain was chosen to rupture the 10% ox-alg semicarbazone and hydrazone gel crosslinks and self-healing recovery was allowed under 1% strain.

Upon rupture, the storage and loss moduli inverted (shown in Figure 3b) and the storage moduli dramatically decreased (<10 Pa). Upon recovery, two-phase self-healing recovery was observed: (i) Rapid bond reformation under 20 s (seconds) upon removing the rupture strain, and (ii) a slower recovery of stiffness observed during the next 160 s. The rapid bond reformation regained a majority of the network stiffness ≈70% (820 Pa out of 1160 Pa) and ≈40% (131 Pa out of 356 Pa) of their initial storage moduli compared to ≈10% and ≈5% in the slower recovery phase for semicarbazone (denoted SemiCarb in Figure 2) and hydrazone crosslinks, respectively. Interestingly, crosslinks recover ≈ 10% stronger after the 2nd and 3rd rupture cycles compared to the 1st rupture cycle. This result can partially be attributed to the inability for reactive chain ends to immediately find each other upon recovery. This kinetically traps the hydrogel in a temporarily less cross-linked state (lower modulus), which then slowly recovers towards the initial modulus after time and the rearrangement induced by subsequent self-healing cycles. The higher number of binding sites in the 15% ox-alg hydrazone gel is reflected in a high moduli recovery after conformational network changes (shown in Figure S7 in Supplementary Materials).

To visualize self-healing macroscopically, two disk shape solid gels with different colors (red and green) were made. For a given linker, each pair of gels was cut into two halves and then put back into contact within five minutes. We observed that the semicarbazone gels self-healed faster than the hydrazone gels and that the oxime gels did not self-heal. After four hours, semicarbazone and hydrazone gels self-healed and the gel boundaries became obscured. Self-healed gels could be stretched using tweezers after 24 h: The semicarbazone gel interface stayed stable under stretching force; however, upon overstretching the hydrazone gel showed cracks across the interface (see in the Figure 3c). The oxime gel did not self-heal at all, even after 24 h (images can be seen in Figure S8a in Supplementary Materials).

#### 2.3.4. Cell Viability

**Figure 4:**
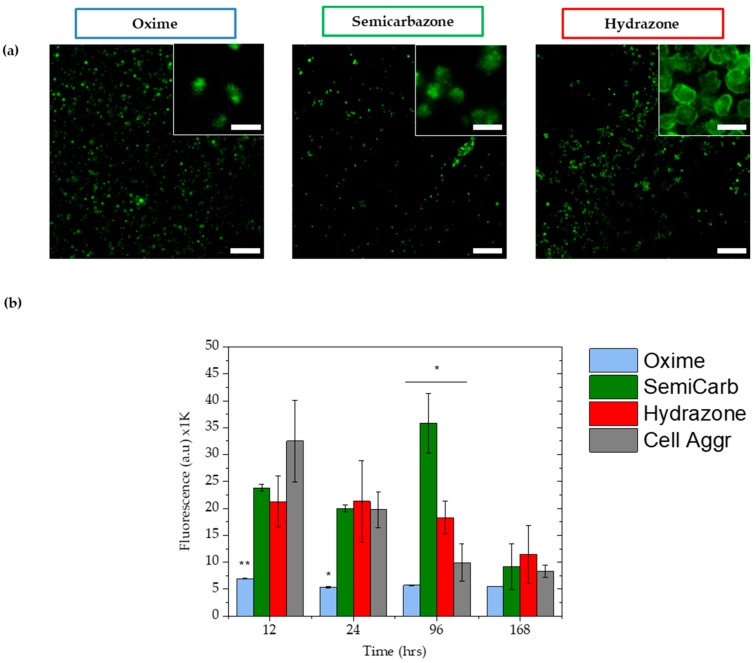
Cell viability: (**a**) Image showing live cells (in green) after seven days of encapsulation in gels indicating that great majority of cells are live and gels did not cause cytotoxicity, Scale bar: 200 µm and 25 µm for inset images. (**b**) Cell metabolic activity recorded after 12, 24, 96, and 168 h. The values reported are an average of *n* = 3, ± standard deviation. * and ** indicates *p* < 0.05 and *p* < 0.01 (Student’s *t*-test, independent sample populations). For cell aggr condition, cells were cultured in pellets, a standard for chondrocyte cell culture.

ATDC5 chondrocytes cells were used for cell viability studies (live-dead and metabolic activity) as they are known to survive in gels without biochemical cues (e.g., RGD, a peptide motif required for cell adhesion to ECM) [48,49] and form multicellular aggregates [50]. ATDC5 were encapsulated (3D) within 10% ox-alg gels and cells were stained and imaged using an inverted fluorescence microscope to evaluate cytotoxicity after one, four, and seven days. Shown in Figure 4a, great majority of cells were found to be alive in all gels after seven days. Live-dead images for one and four days are shown in Figure S9 in Supplementary Materials. The oxime gels were stable over seven days of culture with minimal erosion at the edges. The semicarbazone and hydrazone gels swelled and eroded over time and were found to be very soft/fragile to handle on day seven. Interestingly, over time, the hydrazone gels appeared to facilitate cell clustering (high magnification images are inset in Figure 4a). In the semicarbazone and the oxime gels this clustering of cells was noticeably less, which is hypothesized to be a result of less dynamic reorganization of the network. The high multi-day viability of cells within these gels supports future use for cell culture and bioprinting applications. 

To confirm the high viability for chondrocytes seen during the live/dead assay, a prestoblue assay was run to investigate the metabolic activity of the cells within the gels over 168 h (seven days) (Figure 4b). Within a series of 10% ox-alg, 2% (*w*/*v*), gels, the oxime hydrogels maintained significantly lower metabolic activity compared to semicarbazone and the hydrazone hydrogels, which showed similar metabolic activity to the chondrocyte cell aggregate pellet—the tissue engineering standard for chondrocyte cell culture. In particular, chondrocytes showed significantly different metabolic activity in all conditions on day four. Comparing all gel samples, chondrocytes in the oxime hydrogels showed a consistently low metabolic activity, while other samples started with a high metabolic activity that slowly decreased over seven days. The chondrocytes studied were able to maintain a higher metabolic flux in the more viscoelastic hydrogels (semicarbazone and hydrazone), as compared to the static (oxime) hydrogels.

#### 2.3.5. Cell Spreading

**Figure 5:**
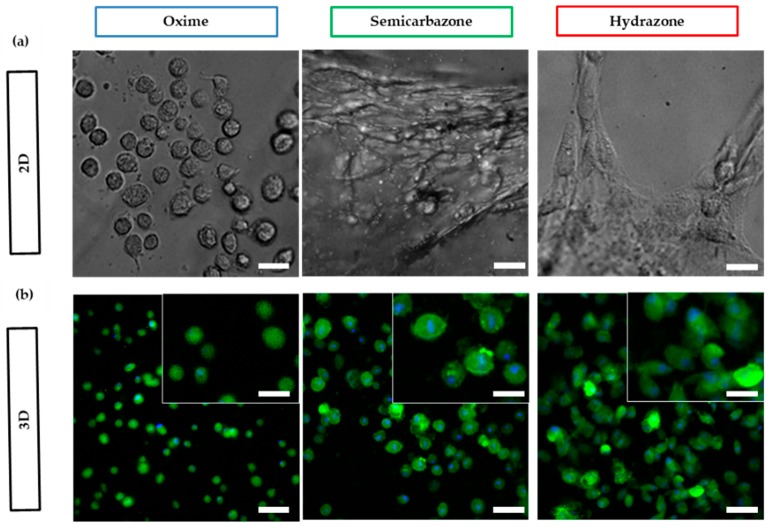
Fibroblasts spreading morphologies in oxime RGD ligated gels, (**a**) on top of (2D) and (**b**) within (3D) oxime, semicarbazone and hydrazone cross-linked gels after 24 h. Green color represents actin staining and blue color represent nucleus staining. scale bar: 25 µm for 2D, 50 µm for 3D images, and 25 µm for 3D insets.

Alginate possesses no active adhesion sites to interact or attach to mammalian cells, but cell adhesion and interaction can be promoted through the conjugation of cell adhesion ligands (e.g., RGD) [42]. Conveniently, the oxidized alginate backbone can readily be ligated with hydroxylamine containing biomolecules via the well-established oxime ligation [51,52]. Hydrogels were bio-functionalized by incorporating aminooxy-RGD ligand (1000 µM) [53] to the 10% ox-alg hydrogel formulation. To investigate whether the crosslinks with different viscoelasticity have an influence on cell spreading, human dermal fibroblasts (HDFs) were seeded on top of gels (2D) for 24 h (see Figure 5). HDFs are better models for cell adhesion and spreading since they are phenotypically adherent and demonstrate an adhesion dependent spreading morphology in 2D cell culture.

We noticed a marked effect on the amount of spreading on the different DCvC cross-linked gels. Cell spreading was found to increase for networks cross-linked by bonds with a higher hydrolysis rate (*k*_−1_), namely, more dynamically rearranging crosslinks. HDFs seeded on the dynamic hydrogels (semicarbazone and hydrazone) showed a spindle shape, increased spreading, and an elongated morphology, with the hydrazone networks showing a more elongated morphology than the semicarbazone. For the oxime hydrogels, only rounded morphologies typical of cells in elastic hydrogels were observed (Figure 5). These results are in accordance with reported *k*_−1_ values of the crosslinks: Hydrazone has highest *k*_−1_, which suggests that crosslinks were permissive to changes in cell shape, while oxime has the lowest *k*_−1_, resulting in elastic gels where limited cell spreading were observed. As a control, cells were seeded on tissue culture plastic (TCP) and exhibited typical spreading morphology (see Figure S10 in Supplementary Materials).

Of note, adherent cells generally increase their spreading area with an increase in substrate stiffness (2D elastic) [54]. This would suggest that cells should actually spread more on the oxime gels (stiffest and most elastic); however, we have observed larger cell spreading on viscoelastic matrices (semicarbazone and hydrazone). The same trend was observed when HDF were cultured within gels (3D). These results are in accordance with recent literature finding a relationship between cellular spreading and reversible hydrogel networks [24,29,33]. Further exploration is warranted, yet our current hypothesis is that microenvironment clustering of adhesive ligands [55], or stress relaxation [29] plays a significant role.

### 2.4. Injectability and Bioprintability

**Figure 6:**
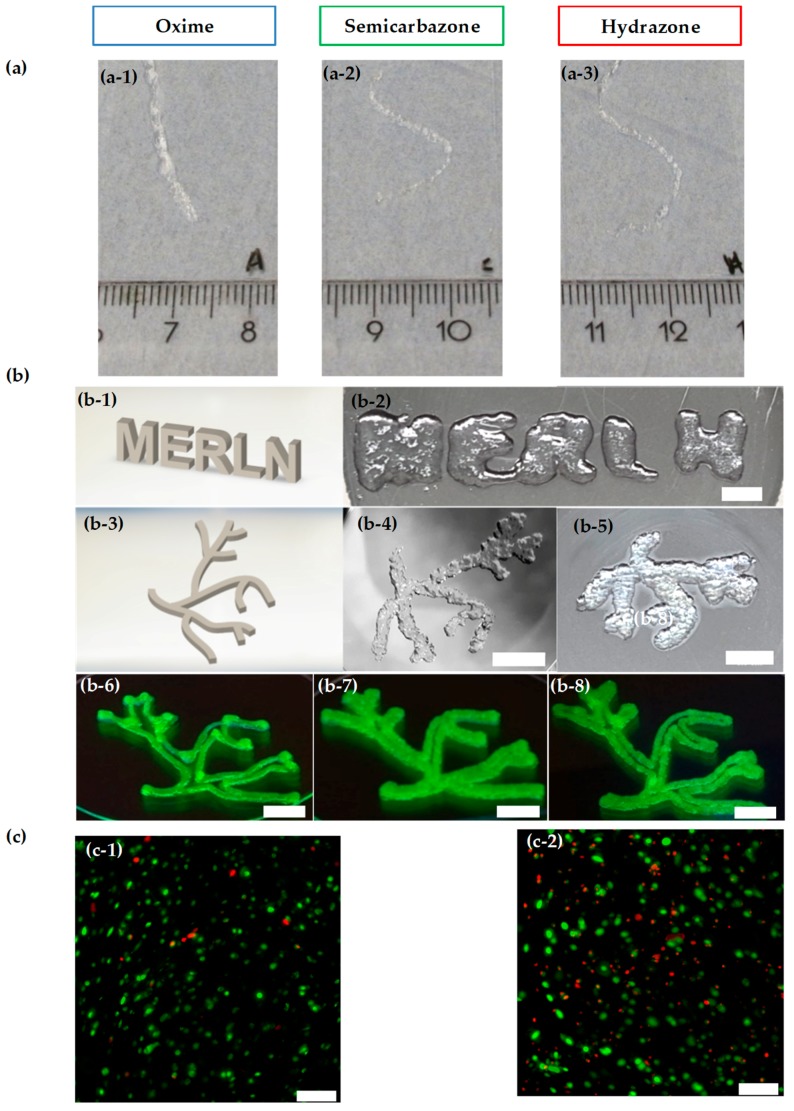
Injectability and bioprintability: (**a**) 10% ox-alg gels with oxime (**a-1**), semicarbazone (**a-2**), and hydrazone (**a-3**) cross-linkers were extruded through a 25G needle. Semicarbazone and hydrazone showed injectability, and to our surprise oxime gels were also injectable, although they were not self-healing. (**b**) Printed MERLN and vascular tree structures using hydrazone crosslinks; 2% (*w*/*v*) of the 10% ox-alg used for (**b-2**–**b-6**) and 3% (*w*/*v*) and 4% (*w*/*v*) of 5% ox-alg used for (**b-7**) and (**b-8**), respectively. From the top left: 3D model of MERLN structure (**b-1**), printed MERLN structure (**b-2**, 6 mm thickness), 3D model of vascular tree (**b-3**), printed vascular tree (**b-4**, 2 mm thickness), printed vascular tree (**b-5**, 6 mm thickness), printed vascular tree (**b-6**–**b-8**) where the network was manually disrupted (fluorescein included for visibility), scale bar: 5 mm. (**c**) 10% ox-alg hydrazone gels after 24 h, (**c-1**): Without printing and (**c-2**): After bioprinting. Green color represents live cells and red color represents dead cells, Scale bar: 200 µm.

With control over the materials properties, self-healing, cell viability, and bio-functionalization in these materials, we next investigated the injectability of these hydrogels. To assess the shear-thinning capability of the 10% ox-alg with different cross-linkers, we attempted to inject these materials from a syringe through a 25 gauge needle (260 μm internal diameter). The hydrazone and semicarbazone hydrogels were injectable through the needle with only the force of the hand and formed intact smooth fibers due to their self-healing capability (Figure 6a). Interestingly, the oxime gel was also found to be injectable, but the injected fiber was a non-continuous gel slurry upon injection. Hydrogels made from the 15% ox-alg with both hydrazone and semicarbazone were also observed to be injectable; however, the 15% semicarbazone hydrogels did not produce smooth fibers and required more force for injection (Figure S11b in Supplementary Materials). These initial experiments encouraged the exploration for use as bioinks for bioprinting and forecast their suitability as drug or cell delivery vehicles.

While both hydrazone and semicarbazone gels showed some initial printability, the semicarbazone gels required significantly higher pressures and larger needle diameters. Consequently, the hydrazone gels were deemed more likely to support cell viability and were further optimized for bioprinting. A simple grid structure was employed to study the effects of deposition speed and extrusion pressure on printability. Using different deposition speeds and extrusion pressures, we used the 10% ox-alg 2% (*w*/*v*) hydrazone hydrogels to deposit 2-layered grid structures via a 0.25 mm diameter conical needle. Extrusion of the hydrogel at 115 and 120 kPa were observed to be more homogeneous and better defined structures were extruded at a speed of 5 mm/s, as opposed to the partial and inhomogeneous hydrogel fibers bioprinted at 7 mm/s (Figure S12 in Supplementary Materials). This could be expected as the bioprinting speeds employed should allow ample time for an appropriate amount of material to be deposited and placed on a substrate. Similarly, extrusion pressure was optimized to tune the amount of deposited hydrogel and three different pressures (115, 120, and 140 kPa) were tested. A pressure of 140 kPa was found to be the optimal pressure for fiber extrusion among the tested values (Figure S13 in Supplementary Materials).

We also tested printability with a lower concentration of the 10% ox-alg (1% (*w*/*v*)) and 5% ox-alg hydrazone hydrogels (2% (*w*/*v*)). Since these formulations form softer and less viscous gels, smaller diameter needles, and low extrusion pressures were tried for printing. Though both formulations showed promising 2-layered constructs, the integrity of the structure was compromised owing to their softer nature (printed structures are shown in Figures S14 and S15 in Supplementary Materials).

Utilizing the optimized pressure (140 kPa) and deposition speed (5 mm/s) for the 10% ox-alg (2% (*w*/*v*)) hydrogels, we bioprinted one, two, and four layered grid scaffolds (Figure S16 in Supplementary Materials). These hydrogels show printability, yet are not able to recreate high-fidelity grid formation as seen in high-viscosity and covalently cross-linked formulations. While grid formation provides good insight into bioprinting fidelity of a grid, our aim to create soft tissue constructs prompted the exploration of either more life-like or solid structures. To investigate the ability of the 10% ox-alg hydrazone gel to make more complex and self-supporting structures, we bioprinted the name of our institute “MERLN”, and a vascular tree model, both with 6 mm thickness (shown as b-2 and b-5 in Figure 6). While the recreation of the vascular tree was found possible, the printing of sharp angels and closely spaced or parallel lines resulted in occlusion and merging as found in the MERLN name.

To increase bioprinting fidelity and print self-supporting structures with continuous deposition of material, printability was also tested by manually disrupting the gel network before printing. Preprinting network disruption allowed uniform deposition of material at seven mm/s using 150 kPa, and this modification resulted in a vascular structure with better printing resolution and uniformity (shown as b-6 in Figure 6). This manual disruption method was also tested using 5% ox-alg (3 and 4% (*w*/*v*) and resulted in a similar result (shown as b-7 and b-8 in Figure 6). However, these higher concentrations of hydrogel (3–4% (*w*/*v*)) needed significantly higher pressure (600 kPa) compared to 2% (*w*/*v*), 10% ox-alg (150 kPa). Further exploration of printing parameters and network dynamics modifications are expected to increase the printability of these materials.

The viability of 10% ox-alg (2% (*w*/*v*)) hydrazone gels with ATDC5 was evaluated 24 h after bioprinting and compared it to the control, without bioprinting (shown in Figure 6c). Very few dead cells were found, which is in agreement with the cell viability experiment carried out over seven days (shown in Figure 4a). Cell viability decreased after 24 h of bioprinting, which could be attributed to the shear stress suffered by cells during bioprinting (c-2 in Figure 6).

## 3. Conclusions

In this study, we have investigated the suitability of hydrogels based on oxidized alginate with imine type crosslinks as an injectable and printable biomaterials platform. The different cross-linkers had a marked effect on gel formation, viscoelasticity, shear-thinning, self-healing, injectability, and printability. All explored formulations were non-cytotoxic over seven days. The viscoelasticity could be tuned by selecting the imine type of crosslinks: Oxime with lowest off rate (*k*_−1_) behaved like an elastic gel and hydrazone with highest off rate (*k*_−1_) behaved like a viscoelastic gel. All cross-linkers resulted in injectable gels, but only the semicarbazone and hydrazone gels are self-healing. Hydrazone crosslinks 10% ox-alg (2% (*w*/*v*)) showed printability of self-supporting structures and high cell viability after bioprinting. HDFs cell studies showed that viscoelastic crosslinks (semicarbazone and hydrazone) encouraged spreading morphology, while elastic crosslinks (oxime) favored rounded morphology and were not permissive to changes in cell shape. These gels represent a step towards smarter viscoelastic materials for 3D bioprinting and bioink formulations. In the future, this platform will be further explored for the effect of materials dynamics on tissue formation and for formulations with improved printability. We envision a smarter bioink that fulfils both the necessities of printability, while greatly improving upon the mimicry of the extracellular matrix.

## 4. Materials and Methods

### 4.1. Materials

Propanoic acid hydrazide (≥90%), *O*-ethylhydroxylamine hydrochloride (97%), adipic acid dihydrazide (≥98%,), *O*,*O*′-1,3-propanediylbishydroxylamine dihydrochloride (98%), propyl isocyanate, hexamethylenediisocyanate, hydrazine monohydrate, activated charcoal (Norit), sodium periodate (NaIO_4_) and ethylene glycol were purchased from Sigma-Aldrich. Propyl semicarbazide and *N*,*N*′-(hexane-1,6-diyl)bis(hydrazinecarboxamide) (HDCA) (^1^H NMR structures shown in Figures S1 and S2 in Supplementary Information) were synthesized according to literature [38]. Sodium alginate was purchased from FMC (Manugel GMB, Lot No. G9402001, Dialysis membrane (Spectra/Por^®^) with molecular weight cut off (MWCO) 3500 Dalton (Da) from VWR, Netherlands. 

Dulbecco’s modified Eagle’s medium (DMEM, high glucose and supplemented with GlutaMAX™ and Sodium Pyruvate), DEME-F12 (low glucose), Dulbecco’s phosphate buffer saline (PBS), fetal bovine serum (FBS), penicillin/streptomycin (P/S), calcein AM, ethidium homodimer, Alexa Fluor^®^ 488 Phalloidin, DAPI and PrestoBlue™ cell viability reagent were purchased from Thermo Fisher Scientific. 

### 4.2. Alginate Purification

Alginate powder was dissolved in deionized (DI) water at a concentration of 1% (*w*/*v*). Activated charcoal (1% (*w*/*v*)) was added, and the alginate solution was stirred for 24 h at 4 °C. Subsequently, the alginate solution was filtered with 11 μm, 1.2 μm, 0.45 μm, and 0.2 μm Whatman membrane filters (Sigma-Aldrich) to remove charcoal particles. The alginate solution was then frozen and lyophilized. 

### 4.3. Synthesis of Oxidized Alginate

To prepare 5% ox-alg, purified alginate (1.00 g, 5.68 × 10^−3^ mol monomer) was dissolved in 100 mL DI water. Keeping the reaction in the dark, sodium periodate (6.07 × 10^−2^ g, 2.84 × 10^−4^ mol) was added in one portion with stirring. After 17 h in the dark (at RT), the reaction was quenched by addition of equimolar ethylene glycol (1.76 × 10^−2^ g, 2.84 × 10^−4^ mol). The reaction solution was stirred in dark for 1 h to stop the reaction completely. The reaction solution was dialyzed against water (2 litre bucket) using membrane tubes with a MWCO of 3500 Da (3 days with 3× water change per day), then lyophilized (over 80% yield). The procedure was repeated to prepare 10% ox-alg and 15% ox-alg.

### 4.4. Preparation of Hydrogels for Rheology and Cell Culture

Oxidized alginate samples were weighed into 1.5 mL Eppendorf tubes. To prepare alginate solutions of 2.5% (*w*/*v*), PBS was added and the solution was mixed for 30 min on a thermoshaker at RT (2000 rpm). Cross-linker solution was added (prepared in PBS) to prepare hydrogels with equimolar concentrations of aldehyde/cross-linker functionalities with a final alginate concentration of 2% (*w*/*v*). Solutions were quickly transferred to polydimethylsiloxane (PDMS) molds with a disc geometry of 12 mm in diameter and 2.0 mm in thickness. Coverslips were placed on top of gel solutions during the gelation process to ensure hydrogels have flat top surfaces and homogeneous thickness. Gelation was left to occur for 30 min at room temperature and then overnight at 4 °C before rheological measurements were carried out. For cell culture studies, the alginate solution was incubated at 37 °C for gelation for 45 min.

For cell spreading, alginate was biofunctionalized with RGD: Oxidized alginate was dissolved in PBS and aminooxy RGD [53] (see Supplementary Materials for synthesis procedure) was coupled to the network using oxime ligation [51]. The final RGD ligand concentration was set to 1000 µM and equimolar concentrations of cross-linkers (relative to aldehyde functionalities) were added and mixed uniformly to form a gel with a final 2% (*w*/*v*) oxidized alginate.

### 4.5. Rheological Measurements

Rheological measurements of hydrogels were carried out using an Anton Paar MRC 702 (Graz, Austria) at 23 °C using a parallel plate geometry with bottom and top diameters of 50 mm and 12 mm, respectively. During loading, the experimental gap size was set when a threshold normal force was reached; 1 N for stiffer gels and 0.1 N for softer gels. This ensures good contact with the plates, prevents slippage, and increases the sensitivity of measurements by increasing the torque response. Samples were protected against evaporation by the addition of 2–3 drops of distilled water to the exposed surface after loading.

Oscillatory strain amplitude sweeps were conducted with strains from 1% to 800% at a frequency of 10 rad/s. Oscillatory frequency sweeps were performed from either 0.1 rad/s or 1 rad/s up to 100 rad/s. Step-strain measurements were undertaken to evaluate the self-healing capacity of hydrogels. Samples were subjected to three cycles, each consisting of 1% strain at 10 rad/s for 180 s, followed by 600% strain at 10 rad/s for 100 s.

### 4.6. Macroscopic Self-Healing Experiment

Macroscopic self-healing behavior of 10% ox-alg hydrogels was evaluated by observing gel recovery at various time points (2, 4, and 24 h without the application of any external pressure. Disc-shaped hydrogels were prepared in the presence of small amounts of food coloring (green and red). The gels were cut down the middle into two pieces with a scalpel and two different colored pieces were placed in contact to determine self-healing behavior. For 15% ox-alg, gels were made in glass vials and the network was broken using a spatula, and then checked every five minutes.

### 4.7. Printability and Injectability 

For printability, alginate solutions with hydrazone cross-linkers in PBS were loaded into 3 mL cartridges with stoppers and were left to form hydrogels for 18 to 20 h at 4 °C before use. For printing, a conical G25 needle was used on a BioScaffolder (GeSiM–Gesellschaft für Silizium-Mikrosysteme mbH, Radeberg, Germany) controlled through proprietary software. Scaffolds with grid geometries were created with settings for a four-corner polygon, comprising 7 meandered strands placed at a distance of 1.0 mm apart. The deposition angle was rotated by 90° after each layer. The height of each layer was set to 0.2 mm and the number of layers was varied accordingly. “MERLN” and vascular tree constructs were designed and drawn in SolidWorks before being converted to .stl files. For manual disruption, 2% (*w*/*v*) of the 10% ox-alg and 3 and 4% (*w*/*v*) of 5% ox-alg with hydrazone cross-linker were left to form hydrogels for 2 to 3 h. Manual disruption was performed by connecting the printing cartridge to a 5 mL syringe with a Luer-lock adapter. The hydrogel was forced between compartments until gel disruption was observed and then extruded through a 25G needle. For injectability, 2% (*w*/*v*) gels for each cross-linker were prepared and extruded through a 25G needle. 

### 4.8. Cell Culture

ATDC5 chondrocytes cells were cultured at 37 °C under a 5% CO_2_ atmosphere in Dulbecco’s Modified Eagle’s Medium-F12 (DMEM-F12, low glucose) supplemented with 10% (*v*/*v*) fetal bovine serum (FBS) and 1% (*v*/*v*) P/S. Cells were washed with PBS, trypsinized (0.05%), centrifuged, and then re-suspended in a 10% ox-alg solution (details in Section 4.4 above). They were subsequently mixed with cross-linker to yield a homogenous mixture of 2% (*w*/*v*) alginate containing 4 × 10^6^ cells/mL. The alginate mixture (50 μL) was transferred to the PDMS mold to form a gel. Gels were transferred to a non-adherent 24-well plate. For cell pellet, cell suspension was transferred to 15 mL falcon tube with curved bottom. Media was added every two days.

HDF were cultured in Dulbecco’s modified Eagle’s medium (DMEM, high glucose) supplemented with GlutaMAX™, Sodium Pyruvate, 10% (*v*/*v*) fetal bovine serum (FBS) and 1% (*v*/*v*) P/S. Cells passaged at 80–85% confluence and were used after passage 6 for cell spreading experiments. For encapsulating cells within gels (3D), cell suspension and cross-linker solution were mixed with alginate to yield a homogenous mixture of 2% (*w*/*v*) alginate containing 4 × 10^6^ cells/mL. The alginate solution (25 µL) was transferred to PDMS mold and incubated at 37 °C for 45 min to form a gel. Gels were then transferred to a 96 flat bottom black well plate for imaging. For 2D (cell seeded on top of gels), 25 µL of the 2% (*w*/*v*) oxidized alginate mixture was transferred to a 96 flat bottom black well plate, centrifuged at 1500 RCF for five minutes to form a uniform layer on the bottom and left to crosslink at RT for 45 min. HDF cells were seeded on top (2D) of the gel with a cell density of 0.1 × 10^6^ cells/gel. As a control, cells were seeded on TCP with 0.025 × 10^6^ cells/well.

### 4.9. Viability Assays

For live-dead assays, cells were stained with calcein AM/ethidium homodimer Live/Dead solution according to the manufacturer’s instructions on days 1, 4 and 7. For the metabolic assay, PrestoBlue solution (10% (*v*/*v*) in DMEM-F12, supplemented with FBS) was added and cells were incubated for 2 h in the dark at 37 °C. At each time point, 100 µL was collected from each well and the fluorescence intensity was recorded using microplate reader at an excitation wavelength of 530 nm and an emission wavelength of 590 nm.

### 4.10. Bioprintability Viability

For cell viability (live-dead) after bioprinting, 10% ox-alg hydrazone hydrogels containing an ATDC5 cell suspension were prepared, as mentioned above. A cell-laden solution was loaded into a 3 mL cartridge and placed at 37 °C for 1 h. The cartridge with a G25 conical needle attached to it was then placed onto a BioScaffolder (GeSiM–Gesellschaft für Silizium-Mikrosysteme mbH, Germany). Bioprinting was carried out to create a four corner polygon with a radius of 2.0 mm, comprising 2 meandered strands placed at 1.0 mm apart. Single layer constructs with a height of 0.2 mm were created. The pressure was set to 140 kPa and extrusion was performed at 5.0 mm/s. Scaffolds were printed in 12-well non-treated cell culture plates. Live-dead viability assay was carried out after 24 h, as mentioned above.

### 4.11. Cell Spreading

Cell spreading after 24 h was visualized in 3D by staining the actin filaments in the cell body with Alexa Fluor^®^ 488 Phalloidin and the cell nucleus with DAPI. Cells were fixed using 4% PFA for 20 min, washed with PBS, and membrane permeabilized with 0.1% (*v*/*v*) Triton X-100. The cells were then stained using (DAPI (1:1000) and Phalloidin (1:40)) solution in PBS for 20 min at room temperature. Stained cells were imaged with an inverted fluorescent microscope.

### 4.12. Statistics

All measurements were done in at least triplicate. Statistical significance was assessed using the two sample Student’s t-test for independent sample populations in OriginLabs^®^ Origin software. Statistical significance values were set at *p* < 0.05 (*) and *p* < 0.01 (**).

## Supplementary Materials

The following are available online at http://www.mdpi.com/2310-2861/4/4/85/s1.
