# Supplementary material for: Viscoelastic Oxidized Alginates with Reversible Imine Type Crosslinks: Self-Healing, Injectable, and Bioprintable Hydrogels
Source: Gels. 2018 Nov 21;4(4):85. doi: 10.3390/gels4040085 (PMC6318581; doi:10.3390/gels4040085)
Supplement: Supplementary file 1 [file gels-04-00085-s001.pdf]

Supporting Information

Viscoelastic oxidized alginates with reversible imine type crosslinks: self-healing, injectable, and bioprintable hydrogels

Shahzad Hafeez [1], Huey Wen Ooi [1], Francis L. C. Morgan [1], Carlos Mota [1], Monica Dettin [2], Clemens van Blitterswijk [1], Lorenzo Moroni [1] *, Matthew B. Baker [1,*]

[1] Department of Complex Tissue Regeneration, MERLN Institute for Technology Inspired Regenerative Medicine, Maastricht University, P.O Box 616, 6200 MD Maastricht, the Netherlands
[2] Department of Industrial Engineering, University of Padua, Padua 35131, Italy
* Correspondence: l.moroni@maastrichtuniversity.nl ; m.baker@maastrichtuniversity.nl;

Materials and Methods:

Synthesis of small model reacting molecule propyl semicarbazide:

A solution of propyl isocyanate (0.2 g, 2.3 x 10^{-3} mol) in acetonitrile (3 mL) was added dropwise to a solution of hydrazine monohydrate (0.7 g, 14.1 x 10^{-3} mol) in acetonitrile (2 mL) at room temperature with vigorous stirring. Following 24 hrs of stirring, acetonitrile was dried *in vacuo* at 30 °C to yield the product as a white waxy solid. ^{1}H NMR (600 MHz, DMSO-d_6) δ 6.80-6.85 (s, 1H), 6.25 – 6.35 (s, 1H), 4.00 (s, 2H), 3.00 (q, 2H), 1.35 – 1.40 (m, 2H), 0.8 – 0.85 (t, 3H)

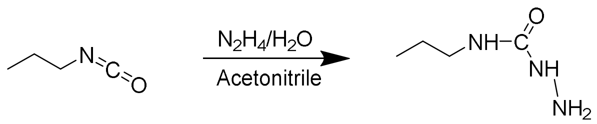

Synthesis of hexamethylene disemicarbazide linker:

A solution of hexamethylenediisocyanate (0.5 g, 2.9 x 10^{-3} mol) in acetonitrile (4 mL) was added dropwise to a solution of hydrazine monohydrate (0.89 g, 17.8 x 10^{-3} mol) in acetonitrile (4 mL) at room temperature with vigorous stirring. Following 2h of additional stirring, acetonitrile was dried *in vacuo* at 30 °C to yield the product as a white solid [38]. ^{1}H NMR (600 MHz, DMSO- d_6) δ 6.75 – 6.85 (s, 1H), 6.29 – 6.21 (s, 1H), 4.00 (s, 2H), 3.00 (q, 2H), 1.32 – 1.40 (p, 2H), 1.26 – 1.20 (m, 2H).

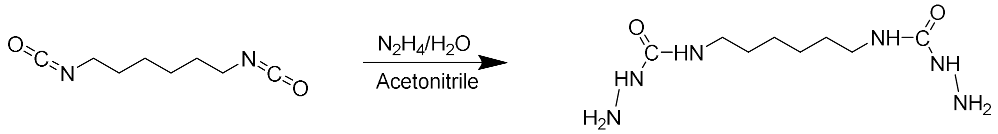

Model reaction with O-ethylhydroxylamine:

Oxidized alginate (10 % ox-alg, 5.00 x 10^{-3} g, 5.05 x 10^{-6} mol of aldehyde groups) was weighed into a 1.5 mL Eppendorf tube. A 0.5 M stock solution of O-ethylhydroxylamine hydrochloride (5.00 x 10^{-3} g, 5.13 x 10^{-5} mol) in D_2O (0.1025 mL) was prepared. The O-ethylhydroxylamine solution (17.70 µL, 8.85 x 10^{-6} mol) and D_2O (0.4823 mL) was added to the tube and the reaction was carried out for 30 minutes on a thermoshaker at RT (2000 rpm). The reaction solution (0.5 mL) was transferred to an NMR tube for recording the NMR spectrum.

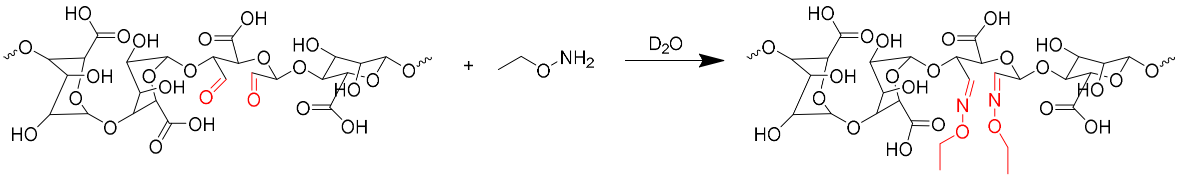

Model reaction with propyl semicarbazide:

Oxidized alginate (10 % ox-alg, 5.00 x 10^{-3} g, 5.05 x 10^{-6} mol of aldehyde groups) was weighed into a 1.5 mL Eppendorf tube. A 0.5 M stock solution of propyl semicarbazide (5.00 x 10^{-3} g, 4.27 x 10^{-5} mol) in D_2O (0.0854 mL) was prepared. The propyl semicarbazide solution (17.70 µL, 8.85 x 10^{-6} mol) and D_2O (0.4823 mL) was added to the tube and the reaction was carried out for 30 minutes on a thermoshaker at RT (2000 rpm). The reaction solution (0.5 mL) was transferred to an NMR tube for recording the NMR spectrum.

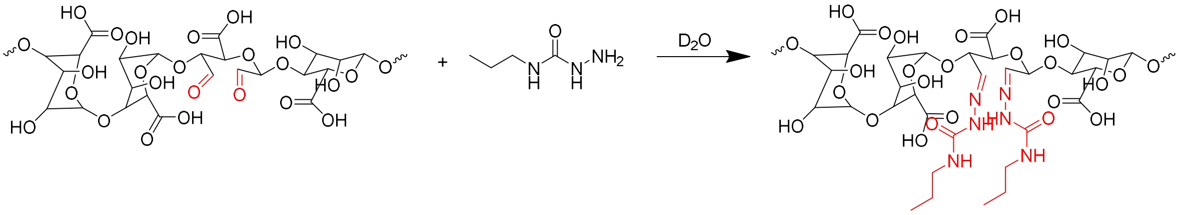

Model reaction with propanoic acid hydrazide:

Oxidized alginate (10 % ox-alg, 5.00 x 10^{-3} g, 5.05 x 10^{-6} mol of aldehyde groups) was weighed into a 1.5 mL Eppendorf tube. A 0.5 M stock solution of propanoic acid hydrazide (5.00 x 10^{-3} g, 5.67 x 10^{-5} mol) in D_2O (0.1135 mL) was prepared. The propanoic acid hydrazide solution (17.70 µL, 8.85 x 10^{-6} mol) and D_2O (0.4823 mL) was added to the tube and the reaction was carried out for 30 minutes on a thermoshaker at RT (2000 rpm). The reaction solution (0.5 mL) was transferred to an NMR tube for recording the NMR spectrum.

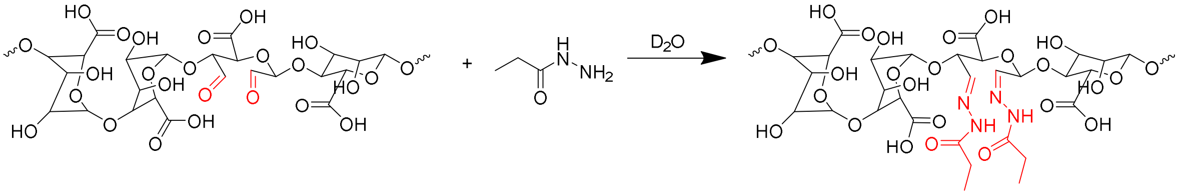

Aoa-7-GRGDSP synthesis:

The peptide Aoa-7-GRGDSP (sequence: NH2-O-CH2-CO-7-aminoheptanoic acid-Gly-Arg-Gly-Asp-Ser-Pro-NH2) was synthesized on Rink Amide MBHA resin (0.60 mmol/g) using Fmoc chemistry by a Syro I synthesizer (Multisyntech, Witten, Germany). The side chain protecting groups were: OtBu, Asp; tBu, Ser; Pbf, Arg. The couplings were double for the first insertion, then single (5 equivalents of Fmoc-amino acid, 5 eq. HBTU, 5 eq. HOBt and 10 eq. DIEA); for the last coupling 10 eq. of 2,4,6-Collidine was used instead of DIEA. The peptide was cleaved from the resin and deprotected from side chain protecting groups using the mixture 1.9 mL TFA, 0.05 mL TES, 0.05 mL H_2O, for 90 minutes. The characterization of the synthetic peptide was performed by MALDI mass spectrometry (Theorethical mass: 786.0 Da; Experimental mass: 784.8 Da; 4800 MALDI-TOF/TOF Analyzer, AB Sciex Pte Ltd, Singapore). The absence in MALDI spectrum of different masses attributable to secondary products encouraged the use of Aoa-7-GRGDSPA without RP-HPLC purification avoiding, on the other hand, amino-oxy group reaction with acetonitrile, the RP-HPLC standard eluent.

Results and discussion:

Synthesis of propyl semicarbazide:

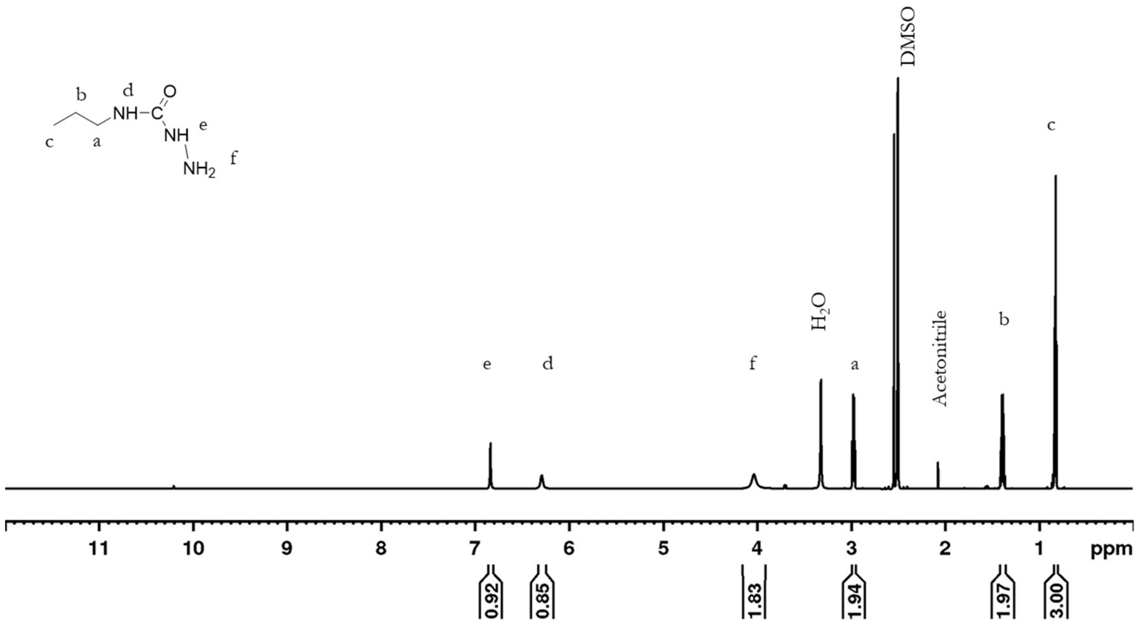

Figure S1: ^{1}H NMR of propyl semicarbazide in DMSO.

Synthesis of hexamethylenedisemicarbazide:

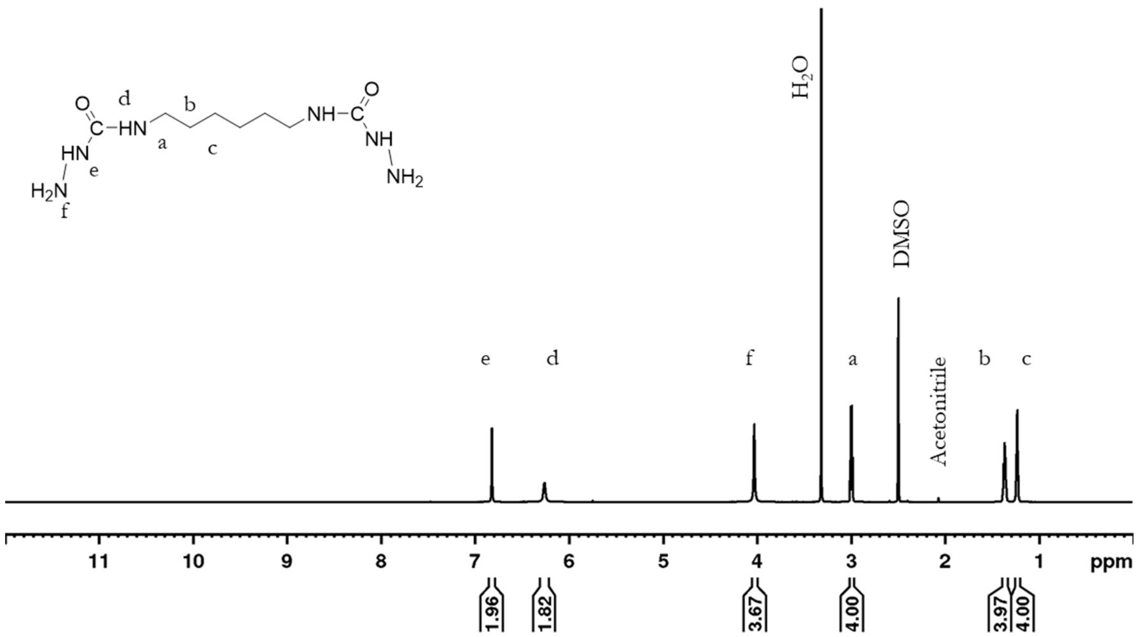

Figure S2: ^{1}H NMR of hexamethylenedisemicarbazide in DMSO.

Oxidized alginate qualitative quantification:

Alginate with aldehyde groups was prepared by oxidation (5%, 10% and 15%) using $NaIO_4$. Oxidation of alginate was confirmed by the presence of hemiacetal peaks in the NMR spectra and it was found that with an increase in the degree of oxidation, the hemiacetal peak intensity increased.

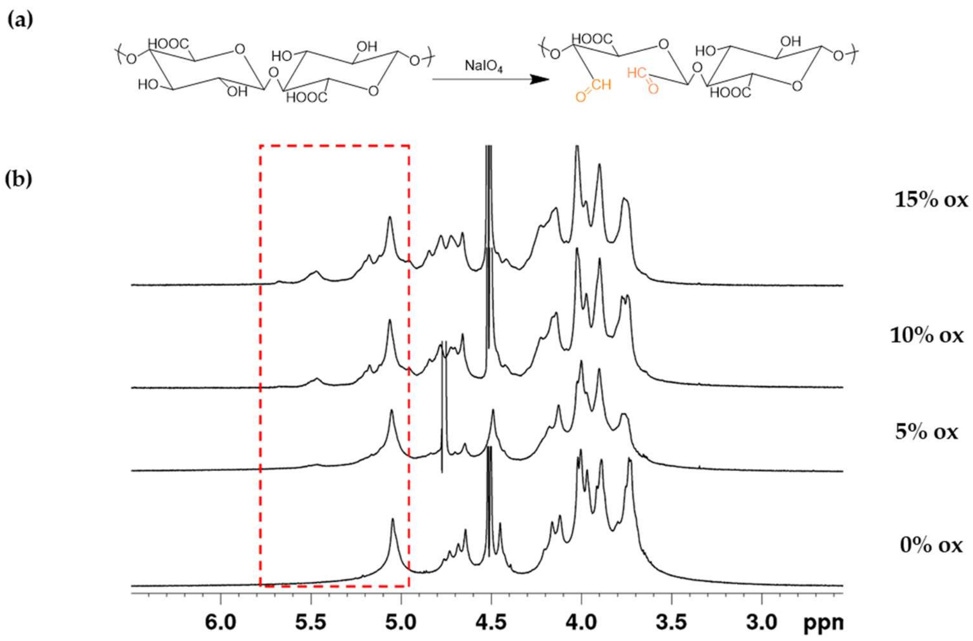

Figure S3: ^{1}H NMR of alginate with varying degrees of oxidation. a) 15%, b) 10%, c) 5%, and d) 0%. The appearance of protons between 5.15 - 5.75 ppm (highlighted in the red box) is attributed to the formation of hemiacetal groups upon reaction of aldehydes to neighboring hydroxyl groups. Spectra were measured at 325 K.

Small molecule model reactions:

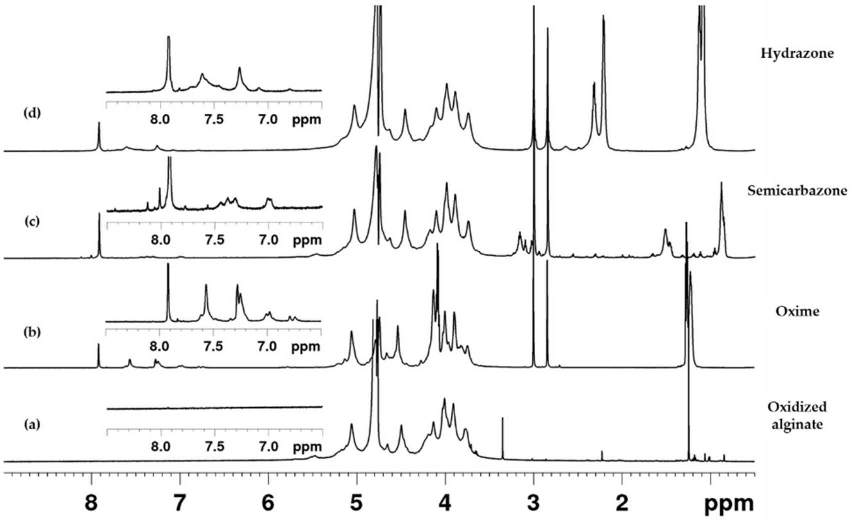

Figure S4: ^{1}H NMR of a) 10% oxidized alginate, b) Reaction solution of 10% oxidized alginate with O-ethylhydroxylamine hydrochloride c) Propyl semicarbazide d) Ethyl hydrazine in D_2O. Protons attributed to the formation of oxime, semicarbazone and hydrazone appear between 6.50 - 8.50 ppm and are highlighted as zoomed in images.

Typical Viscoelastci curve for viscoelastic material:

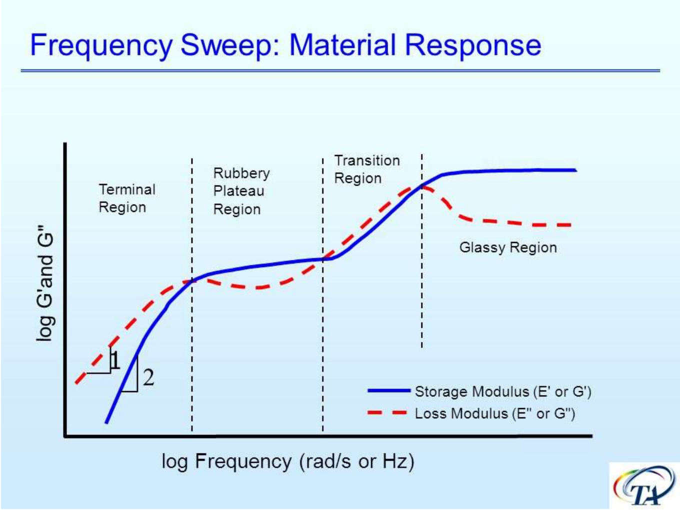

Figure S5: Typical viscoelastic material curve. Reprinted from with permission from TA instruments.

Strain sweeps:

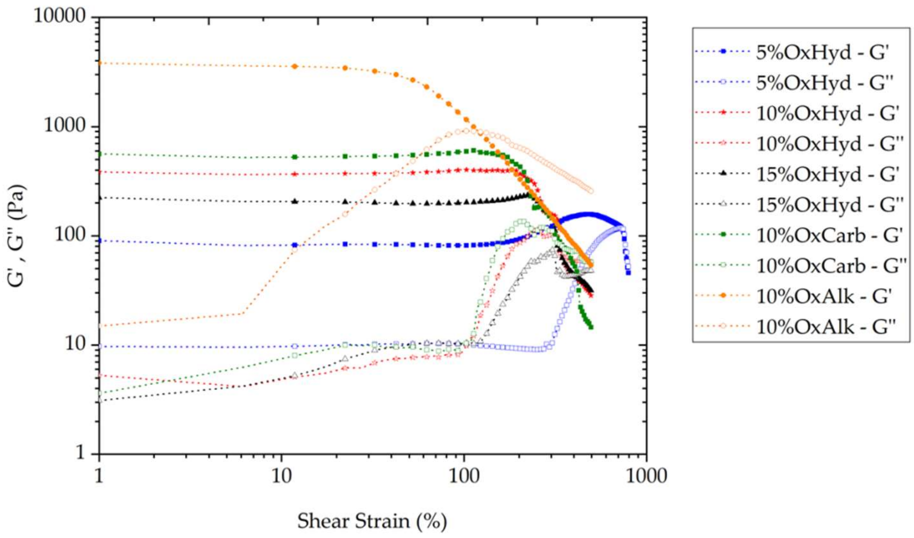

Figure S6: Strain sweeps were carried out to determine the linear viscoelastic region and the critical strain necessary for the onset of network rupture (tanδ = 1). The linear viscoelastic region was shown to be around 100% for most gels, with the exception of the oxime at 10% at 5% hydrazone at 300%. The critical strain for the onset of network rupture was determined to be around 400% with the exception of 10% oxime and 5% hydrazone showing 200% and 800% respectively. Based on these results, 600% strain was chosen for self-healing measurements to ensure network rupture, while 1% strain was chosen for frequency and time sweep measurement to remain within the linear viscoelastic region. While 600% strain does not ensure network rupture for the 5% hydrazone going beyond this point induces such large movements and sample deformation that the sample no longer remains between the parallel plates.

Effect of degree of oxidation

As it can be seen in strain sweep in (figure S6 in SI), that 5% ox-alg network did not rupture completely at 600% strain. Therefore, 5% ox-alg did not follow dynamics of 10% ox-alg and 15% ox-alg. Reliable measurements at a strain sufficient to rupture the 5% hydrazone network (>1200%) were not possible with our parallel plate geometry as the samples were displaced out from under the plates.

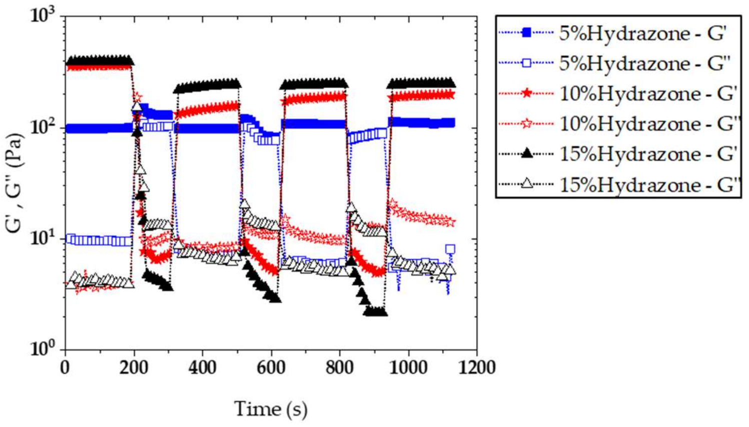

Figure S7: Effect of degree of oxidation on gel storage and loss modulus and self-healing behavior.

Self-healing:

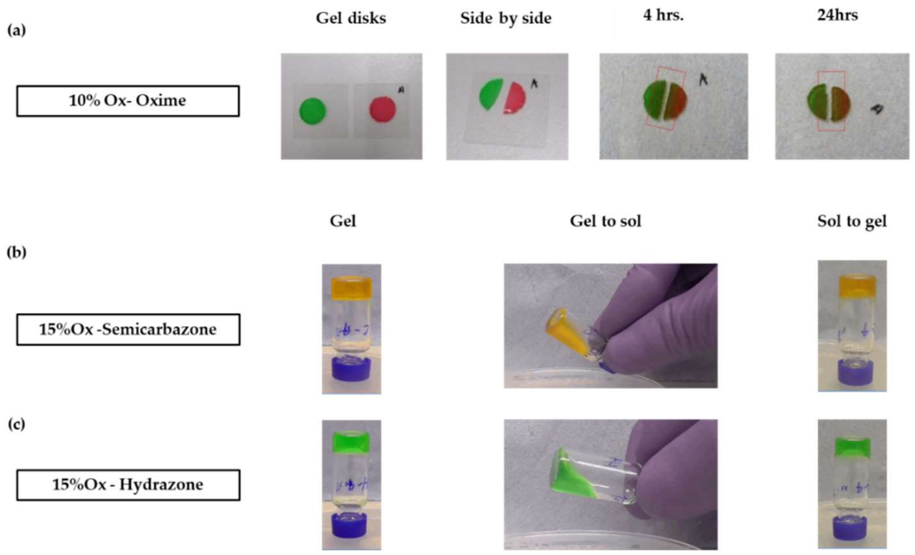

Figure S8: Macroscopic self-healing behavior of (a) 10% ox alginate gel with oxime crosslinks and 15% ox alginate gel with (b) Semicarbazone and (c) Hydrazone crosslinks.

Cell viability

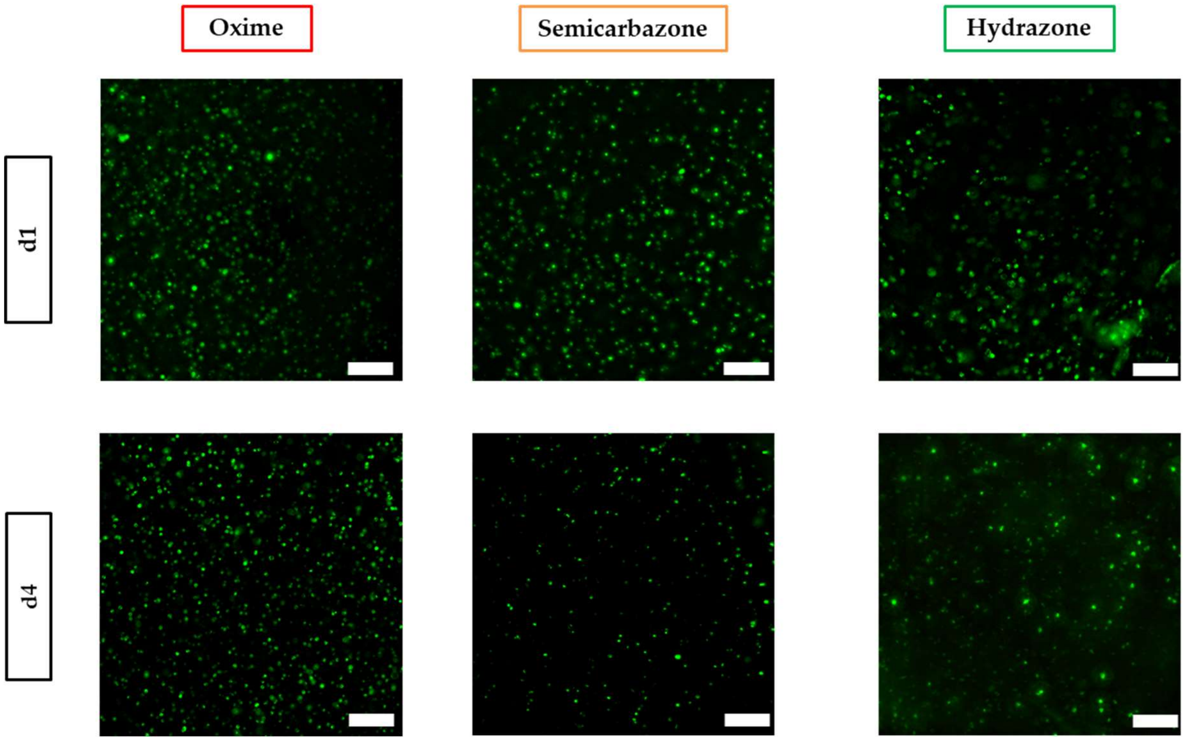

Figure S9: Live-dead staining images of chondrocytes, ATDC5, at 1 and 4 days. Scale bar: 200 µm

Cell spreading:

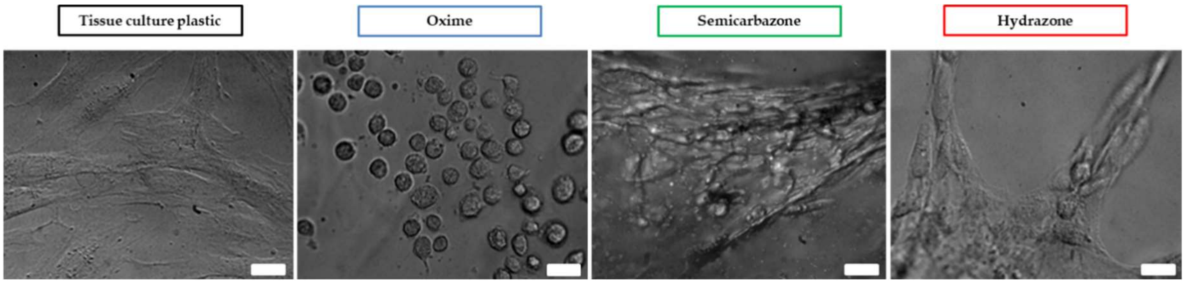

Figure S10: Cell spreading on tissue culture plastic and different imine type reversible crosslinks

Injectability:

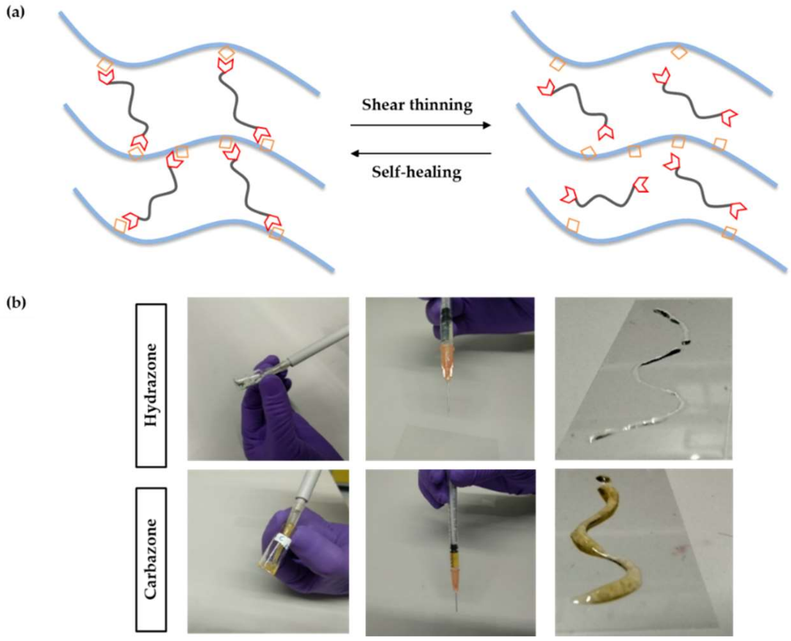

Figure S11: (a) Schematics of injectability for (b) 15% ox-alg gels was also injected using a 25G needle: both hydrazone and semicarbazone were found to be injectable, however semicarbazone did not produce a smooth fiber.

Printability:

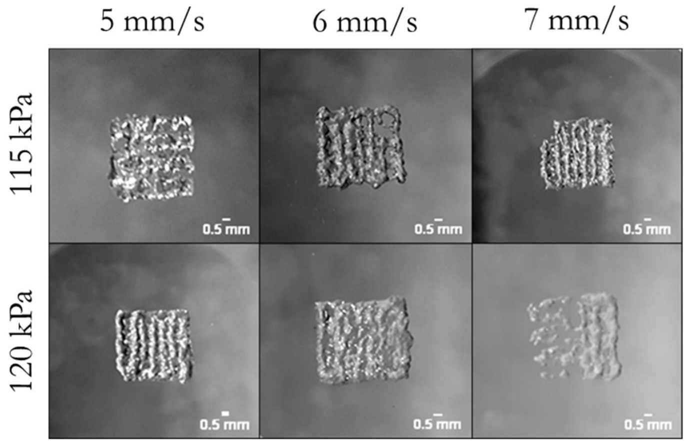

Figure S12: 2-layered scaffolds printed using 10% ox-alg (2% (w/v) alginate) with hydrazone crosslinks printed at different speeds and pressures. A 0.25 mm (25G) conical needle was used.

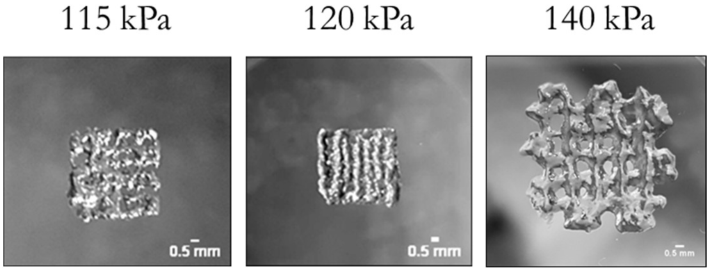

Figure S13: 2-layered scaffolds printed using 10% ox-alg (2% (w/v) alginate) with hydrazone crosslinks printed at 5 mm/s using different pressure values. A 0.25 mm (25G) conical needle was used.

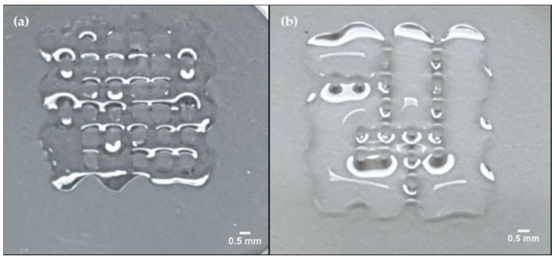

Figure S14: 10% ox-alg (1% (w/v) alginate) hydrazone crosslinked scaffolds of (a) 2 layers and (b) 4 layers printed at 45 kPa (30 mm/s). A 0.20 mm (27G) conical needle was used.

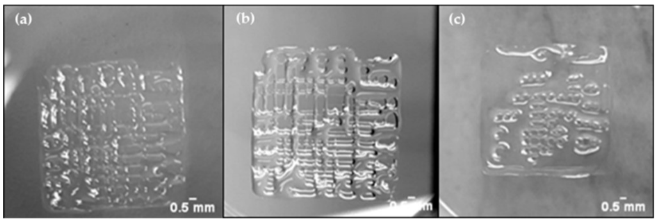

Figure S15: 2-layered scaffolds printed using 5% ox-alg (2% (w/v) alginate) with hydrazone crosslinks at (a) 45 kPa (10 mm/s), (b) 50 kPa (10 mm/s), and (c) 60 kPa (11 mm/s). A 0.20 mm (27G) conical needle was used.

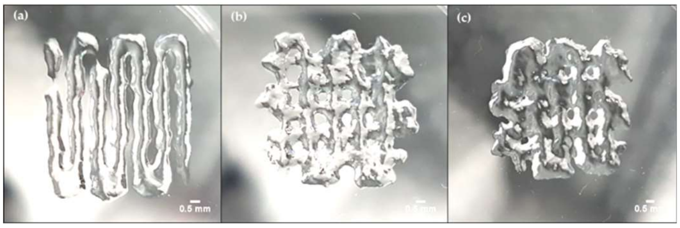

Figure S16: 10% ox-alg (2% (w/v) alginate) hydrazone crosslinked scaffolds of (a) 1 layer, (b) 2 layers, and (c) 4 layers printed at 140 kPa (5 mm/s). A 0.25 mm (25G) conical needle was used.
